# High anti-Mullerian hormone level is adversely associated with cumulative live birth rates of two embryo transfers after the first initiated cycle in patients with polycystic ovary syndrome

**DOI:** 10.3389/fendo.2023.1123125

**Published:** 2023-06-14

**Authors:** Nianjun Su, Juanxiao Zhan, Meiling Xie, Ying Zhao, Cuiyu Huang, Songlu Wang, Liujun Liao, Xiqian Zhang, Fenghua Liu

**Affiliations:** ^1^ Department of Reproductive Health and Infertility, Guangdong Province Women and Children Hospital, Guangzhou, China; ^2^ The First Clinical Medical School of Guangzhou University of Chinese Medicine, Guangzhou, China; ^3^ Department of Gynecology, First Affiliated Hospital of Guangzhou University of Chinese Medicine, Guangzhou, China

**Keywords:** anti-Mullerian hormone, polycystic ovary syndrome, embryo transfer, live birth, pregnancy outcome, assisted reproductive technology, fitting curve

## Abstract

**Objective:**

Anti-Mullerian hormone (AMH) has been recently identified as a potential predictor of live birth rates (LBRs) following assisted reproductive technology (ART) treatment. This study aimed to investigate the association between AMH levels and the outcomes of *in vitro* fertilization (IVF) in patients with polycystic ovary syndrome (PCOS).

**Methods:**

Patients with PCOS initiating their first ovarian stimulation under the gonadotropin-releasing hormone antagonist protocol at the Guangdong Women and Children Hospital, China, were enrolled from November 2014 to September 2018. A total of 157 patients who underwent fresh embryo transfer (ET) cycles were included in group A, whereas 187 patients who underwent frozen–thawed ET cycles were included in group B. After the failure of the first ET cycle, 94 patients underwent the second ET cycle with frozen–thawed embryos. Of these 94 patients, 52 had failed the first fresh ET cycle (group C) and 42 had failed the first frozen–thawed ET cycle (group D). Successful embryo transfer was defined as live birth. This retrospective cohort study addressed the association between AMH levels and pregnancy outcomes using logistic regression approaches. After adjusting for age, body mass index, antral follicle counts, baseline follicle-stimulating hormone levels and baseline progesterone levels, LBRs were compared among the four groups and the cumulative live birth rate after two embryo transfers (TCLBR) was calculated.

**Results:**

The LBRs showed no differences among the four groups. Higher serum AMH levels were found to be associated with a lower TCLBR [adjusted OR 0.937 (0.888–0.987), *P = 0.015*]. In patients who underwent the second ET cycle, LBRs were inversely proportional to AMH levels [crude OR 0.904 (0.828–0.986), *P = 0.022* versus adjusted OR 0.845 (0.754–0.946), *P = 0.004*, respectively]. In addition, the LBR was approximately 61%–78% lower in the group with AMH levels of >12 ng/mL [crude OR 0.391 (0.168–0.912), *P = 0.030* versus adjusted OR 0.217 (0.074–0.635), *P = 0.005*, respectively].

**Conclusions:**

Among PCOS patients high AMH level (>12 ng/ml) is found to be associated with low TCLBR and low LBR of the second embryo transfer cycles. The results provide limited clinical inferences and warrant further investigation.

## Introduction

The prevalence of polycystic ovary syndrome (PCOS) is 4%–21% globally (1) and 7.8% in China (2). Some patients with PCOS undergo *in vitro* fertilization/intracytoplasmic sperm injection (IVF/ICSI) for the treatment of infertility caused by ovulation disorder. PCOS not only manifests as an ovulatory disorder but also may be accompanied by complex gynecological endocrine alterations and may impact the outcome of assisted reproductive technology (ART). Therefore, it is important to identify predictors for pregnancy outcomes of IVF/ICSI.

Anti-Mullerian hormone (AMH), a glycoprotein synthesized by granulosa cells of small follicles in the female ovary, can inhibit the maturation of small follicles (3), demonstrating superiority in predicting ovarian reserve and stimulation responsiveness (3–5). Some studies have suggested the role of AMH levels in predicting the live birth rate (LBR) after IVF/ICSI treatment considering the close relationship between AMH and LBR (6). In addition, scholars have compared the efficiency of follicle-stimulating hormone (FSH) levels, antral follicle counts (AFCs) and AMH levels in predicting live birth. High AMH levels in the same AFC quartile have been showed to associate with a higher cumulative live birth rate (CLBR) and an increased number of oocytes (7). Although the focus of their study was slightly different, Ligon et al. suggested that the predictive efficiency of AMH was superior to that of FSH and revealed that lower AMH levels were independently associated with lower LBRs and increased canceled cycles (8). However, some studies have challenged the capability of AMH in predicting live births after IVF (9, 10), arguing the poor accuracy of AMH in predicting the LBR and clinical pregnancy rate (CPR) in patients undergoing treatment with ART (11–13).

It is not clear yet that AMH associates with LBR or other pregnancy manifestations such as CLBR, increased risk of early miscarriage during initial IVF/ICSI treatment, etc. (14, 15). Some studies have demonstrated that CLBR decreases with an increase in AMH levels (16), especially when AHM levels exceed 5–7 ng/mL (17), whereas other studies have reported that AMH has limited predictive accuracy (18, 19).

A stratified analysis may serve as a more reasonable testing method (20, 21), as both AMH levels and LBRs vary across diseases. AMH levels are lower in patients with diminished ovarian reserve (DOR) but 2–4 times higher in patients with PCOS than those without PCOS (5). The inconsistent results of previous studies have suggested further investigations of the relationship between AMH and pregnancy outcomes after IVF/ICSI in patients with PCOS.

Given that patients with PCOS are predisposed to ovarian hyperstimulation syndrome (OHSS) (22), some ART centers dismiss fresh embryo transfer (ET) for all patients with PCOS, which may inevitably lead to prolonged live birth time and anxiety. Therefore, comparing pregnancy outcomes after fresh and frozen–thawed ET cycles in patients with PCOS is necessary. Most women with PCOS proceed with a second ET cycle after the failure of the first cycle. However, a third attempt after the failure of the second cycle is sporadic. Therefore, evaluating the CLBR after two ET cycles may benefit pregnancy outcomes in clinical settings. In this study, CLBR after two ET cycles was referred to as TCLBR. We analyzed the IVF/ICSI cycle data of patients with PCOS to detect the association between AMH levels and pregnancy outcomes, particularly LBR and TCLBR, under different ET strategies.

## Materials and methods

### Study design and participants

**Figure 1:**
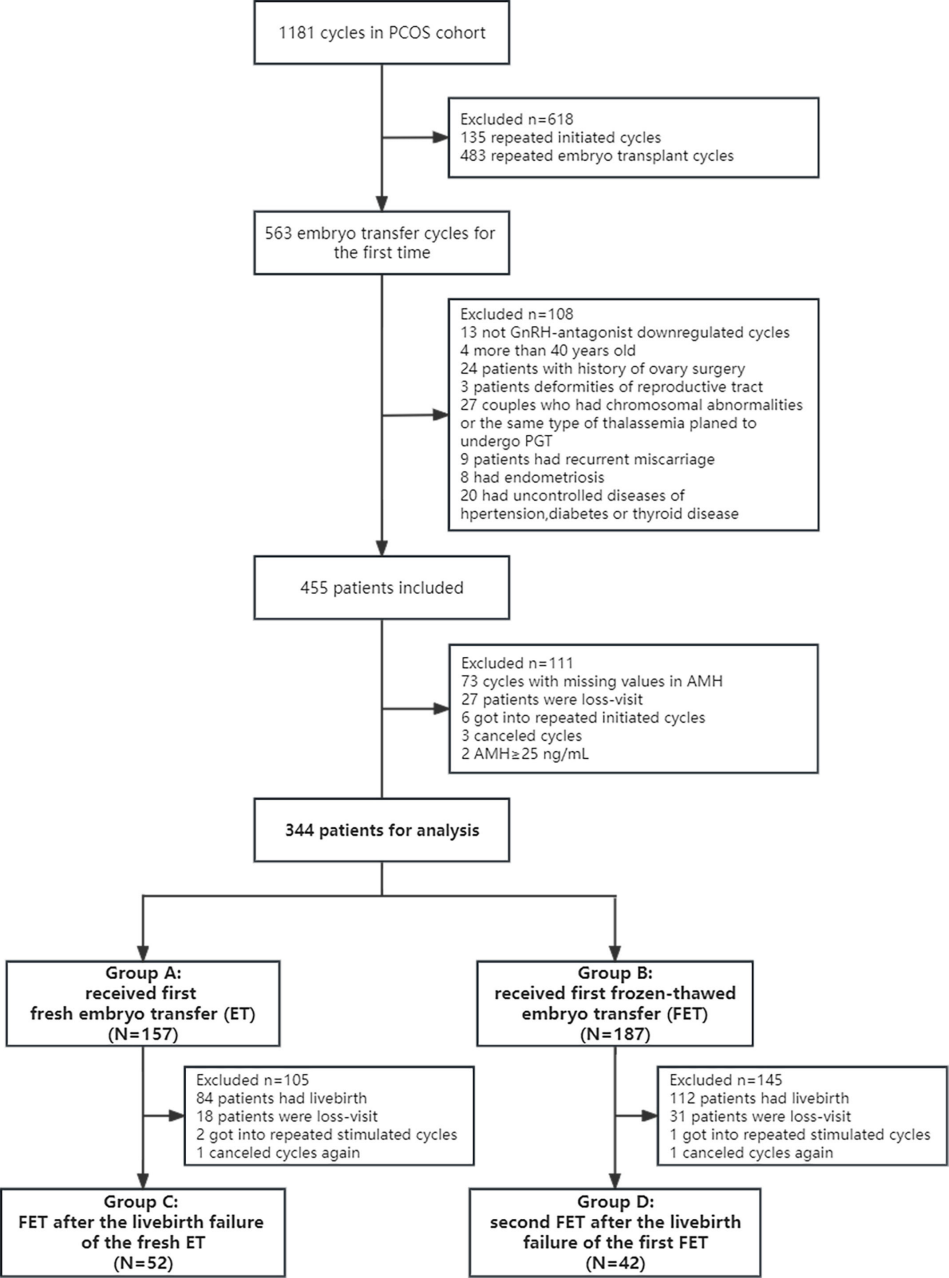
Research flowchart of women with PCOS. Group A: Patients who underwent the first fresh embryo transfer. Group B: Patients who underwent the first frozen–thawed embryo transfer. Group C: Patients who underwent frozen–thawed embryo transfer after the failure of the first fresh embryo transfer. Group D: Patients who underwent frozen–thawed embryo transfer after the failure of the first frozen–thawed embryo transfer. Patients in all four groups were in their first initiated cycle. PCOS, polycystic ovary syndrome; PGT, preimplantation genetic testing; AMH, anti-Mullerian hormone; ET, embryo transfer; FET, frozen–thawed embryo transfer.

This retrospective cohort study enrolled 1181 patients with PCOS undergoing ET cycles at the Reproductive Health and Infertility Department, Guangdong Women and Children Hospital, China, from November 2014 to September 2018. PCOS was diagnosed based on the Rotterdam criteria (23). The exclusion criteria were as follows: repetitive initiated cycles; treatment without the gonadotropin-releasing hormone (GnRH) antagonist protocol; the age of <18 or ≥40 years; history of ovarian surgery; congenital or acquired reproductive malformations; recurrent miscarriages; endometriosis and uncontrolled hypertension, diabetes and thyroid diseases. The initiated cycle was defined as a cycle in which a woman receives specific medication for ovarian stimulation and attempts follicular aspiration. In addition, patients with canceled cycles, those with missing data or with outliers in AMH values and those lost to follow-up were excluded. Canceled cycles were defined as uncompleted ETs after initiating ovarian stimulation. Couples with chromosomal abnormalities or same type thalassemia were excluded, whereas those using donor sperm because of the chromosomal abnormalities of husbands were included. Eventually, 344 patients with PCOS undergoing the first ET in their first initiated cycle under a GnRH antagonist protocol were included. Of these 344 patients, 157 patients undergoing the first fresh ET were included in group A and 187 patients undergoing the first frozen–thawed ET were included in group B. After the failure of the first ET cycle, 94 patients underwent the second ET cycle with the remaining frozen embryos. Patients who underwent the second ET cycle with frozen–thawed embryos after the failure of the first fresh ET cycle were included in group C. Patients who underwent the second ET cycle with frozen–thawed embryos after the failure of the first frozen–thawed ET cycle were included in group D (**Figure 1**). This study was approved by the Guangdong Women and Children Hospital Institutional Review Board, and informed consent was waived.

### Treatment protocol

According to the GnRH antagonist protocol, gonadotropin was injected daily in all patients from the first to the fourth day of their menstrual cycles, irrespective of natural or artificial cycles. Gonadotropin stimulation was performed using recombinant follicle-stimulating hormone (rFSH) (Gonal-F, Merck Serono, Italy, or Puregon, Organon, Oss, the Netherlands) or highly purified urinary FSH (Menopur, Ferring Pharmaceuticals Ltd, Denmark) and combined with human menopausal gonadotropin (HMG; Zhuhai Lizhu Medicine Ltd, China). The initiating dose of gonadotropin varies between 75 and 250 units per day according to age, AFCs, AMH levels, body mass index (BMI) and clinicians’ verdicts. Follicular development was monitored via transvaginal ultrasonography according to serum sex hormone levels. Within 4 days of initiating ovarian stimulation, the dose of gonadotropin was adjusted based on the ovarian response. When serum luteinizing hormone levels decreased below 1.0 IU/mL, 75 IU of injectable HMG or recombinant LH (Luveris, Merck Serono) was administered daily. Ganirelix acetate (ganirelix acetate injection; Organon, Netherlands) or Cetrotide (cetrorelix acetate injection; Merck Serono, Italy) at a dose of 250–500 μg was administered from the fifth day of stimulation (fixed protocol) or when the mean diameter of dominant follicles reached 12 mm (flexible protocol) until the day of triggering.

Ovulation was considered triggered after the diameter of two or more follicles was ≥18 mm, that of at least three follicles was ≥17 mm or that of at least 60% of follicles was ≥15 mm. Human chorionic gonadotropin (hCG, Zhuhai Lizhu Medicine Ltd) at the dose of 8,000 or 10,000 IU or recombinant chorionic gonadotropin (Ovidrel, Merck Serono) at the dose of 250 mg was administered for inducing ovulation. If serum estrogen (E_2_) levels were ≥5,000 pg/mL, the GnRH agonist triptorelin (Gonapeptyl, Ferring) at the dose of 0.2 mg or triptorelin combined with 2,000 IU of urinary hCG was administered to reduce the risk of OHSS. Transvaginal oocyte retrieval was performed under ultrasound guidance within 36 hours of ovulation triggering. Fertilization was accomplished via IVF, ICSI or both. Before July 2017, a self-prepared culture medium was used, which was subsequently replaced with Kato culture medium.

During treatment, the first ET, whether using fresh embryos or frozen–thawed embryos, was performed based on indicators such as fertilization, E_2_ levels after oocyte retrieval, ascites, endometrial condition and patient discomfort. A maximum of two cleavage-stage embryos or blastocysts were transferred. According to the standard protocol, an assessment system was employed for monitoring the morphology of oocytes and early embryos during the culture. Cleavage-stage embryos with at least five blastomeres were considered transferable, and those with 6–10 blastomeres were considered high-quality embryos (at least six cells in an embryo with a maximum of 20% fragmentation on day 3). Blastocysts were assessed according to the Gardner criteria (24): blastocysts with a grade of 3BB and higher were considered high quality.

Embryos were frozen if the risk of OHSS was high or upon patient’s requested. Frozen–thawed ET was performed when endometrial thickness reached 0.8 cm after natural or HMG-induced ovulation or during artificial hormone replacement cycles. As luteal support from the day after oocyte recovery, progesterone or organic hormone was administered as an intravaginal capsule (800 mg per day), or progesterone was administered as a gel (90 mg per day) or via intramuscular injection (40 mg per day). Serum estradiol and progesterone levels were evaluated after 4 days of ET. If E_2_ levels were <200 pg/mL or progesterone levels were <20 ng/mL, estradiol or progesterone was respectively administered. If the pregnancy test indicated positive results, luteal support was maintained until 8 weeks of gestation.

### Outcomes

The primary outcomes included LBR (the ratio of the number of live births to the number of embryo transfer cycles) and TCLBR (the ratio of the total number of live births after the first two embryo transfers to the number of patients who underwent the first initiated cycle). A live birth was defined as the successful delivery of a live baby. The 2017 American Society for Reproductive Medicine (ASRM) international glossary was referenced to define the terms of ART (25), and the Golan criteria were used for classifying OHSS (26). Pregnancy outcomes were monitored through follow-up.

### Statistical analysis

Based on the Shapiro–Wilk test, identified variables with normal distribution were expressed as the mean ± standard deviation, whereas other variables were expressed as the median and interquartile range. In addition, character variables were expressed as the number of counts and percent. The chi-square test was applied for categorical variables and a t-test was applied to continuous variables. Groups A and C represented two cycles of the same patient. Therefore, they could not be compared with the assumption of independence. And the comparison couldn’t be conducted between group B and group D because they were two cycles from the same patient too. We can compare the LBR of group A with group B, group C with group D, and group B with group C. The comparison of the LBR of group A with group D is unsuitable, because group A is the first fresh ET but group D is the second FET. The correlation of AMH levels with LBRs and TCLBRs in the four groups was evaluated via curve fitting. For comparison, patients were grouped according to the turning point of the curve (AMH levels, 12ng/mL). After adjusting for age, BMI, AFCs, baseline FSH levels and baseline progesterone levels, logistic regression analysis was performed to examine the correlation between AMH levels and LBRs in the four groups independently and that between AMH levels and TCLBRs of all 344 patients. Statistically significant differences were indicated by odds ratio (OR) with 95% confidence intervals (95% CIs) of <1, α values of 0.05 (bilateral test) and *P*-values of <0.05. A multivariate logistic regression model based on the generalized additive model was employed to fit the splines. All statistical analyses were performed using the R (version 3.3.2) software package (http://www.R-project.org, The R Foundation) and the Free Statistics (version 1.7) software.

## Results

### Participant’s characteristics

Data regarding the baseline characteristics, ovarian stimulation, implantation and pregnancy outcomes of all 344 patients are summarized in **Table 1**, with AMH levels of 12 ng/mL as the subgroup cut-off value. The number of patients with AMH levels of >12.0 ng/mL was significantly higher in group B (96/187, 67.1%) than in group A (47/157, 32.9%) *(P< 0.001*). Age and the type, duration and cause of infertility were not significantly different between subgroups. Significant differences were observed in baseline variables, including BMI, AFCs, LH levels, LH/FSH ratio, E_2_ levels, prolactin levels, testosterone levels, homeostasis model assessment of insulin resistance (HOMA-IR) levels and fasting insulin levels (*P < 0.05*). In addition, some variables associated with ovarian stimulation and implantation, such as the dose of gonadotropin, number of oocytes retrieved, endometrial thickness on the transfer day, embryo stage and embryo transfer strategies, differed between subgroups (*P <0.05*).

**Table 1:** The characteristics of baseline, ovarian stimulation, implantation, and clinical pregnant outcome of all patients.

Variables	1 ng/mL ≤ AMH < 12 ng/mL	12 ng/mL ≤ AMH < 25 ng/mL	*P*-value
All participants	201	143	
Number of the first transfer			< 0.001
Group A	110 (54.7)	47 (32.9)	
Group B	91 (45.3)	96 (67.1)	
Number of the second transfer			0.202
Group C	34 (60.7)	18 (47.4)	
Group D	22 (39.3)	20 (52.6)	
Age of female patients (years)	29.0 (26.0, 32.0)	29.0 (27.0, 32.0)	0.451
Infertility type			0.918
Primary	124 (61.7)	89 (62.2)	
Secondary	77 (38.3)	54 (37.8)	
Infertility duration (years)	3.0 (2.0, 5.0)	3.0 (2.0, 5.0)	0.403
Infertility cause			0.259
Fallopian tube factor + PCOS	78 (38.8)	61 (42.7)	
Male factor + PCOS	44 (21.9)	30 (21.0)	
PCOS only	62 (30.8)	33 (23.1)	
Compound factor	17 (8.5)	19 (13.3)	
BMI (kg/m^2^)	22.8 (20.4, 25.0)	21.6 (19.9, 23.7)	0.002
AMH (ng/mL)	7.9 (6.1, 9.6)	16.0 (13.5, 18.5)	< 0.001
AFC	24.0 (19.0, 24.0)	24.0 (24.0, 25.0)	< 0.001
Baseline FSH (mIU/mL)	6.1 (5.3, 7.2)	6.0 (5.2, 6.9)	0.471
Baseline LH (mIU/mL)	6.7 (4.9, 10.0)	8.7 (6.2, 12.5)	< 0.001
Baseline LH/FSH	1.1 (0.8, 1.5)	1.4 (1.1, 2.0)	< 0.001
Baseline E_2_ (pg/mL)	34.0 (26.0, 44.0)	38.1 (31.1, 47.0)	0.007
Baseline P_4_ (ng/mL)	0.5 (0.3, 0.7)	0.5 (0.3, 0.7)	0.419
Baseline PRL (ng/mL)	16.7 (12.1, 22.0)	18.7 (13.6, 24.8)	0.037
Baseline T (ng/mL)	0.3 (0.2, 0.4)	0.4 (0.3, 0.5)	< 0.001
HOMA-IR	2.3 (1.6, 3.0)	1.8 (1.4, 2.5)	0.003
FPG (mmol/L)	5.06 ± 0.44	5.01 ± 0.41	0.265
FIns (mU/L)	10.3 (7.0, 13.7)	8.3 (6.2, 11.4)	0.010
FSH levels on starting day (mIU/mL)	6.23 ± 1.44	6.27 ± 1.65	0.833
LH levels on starting day (mIU/mL)	6.7 (4.6, 9.7)	8.2 (5.7, 12.2)	< 0.001
LH/FSH ratio on starting day	1.1 (0.8, 1.5)	1.3 (1.0, 1.9)	< 0.001
E_2_ levels on starting day (pg/mL)	34.1 (24.3, 45.2)	39.0 (30.8, 49.1)	0.005
P_4_ levels on starting day (ng/mL)	0.4 (0.2, 0.5)	0.4 (0.2, 0.6)	0.102
Duration of stimulation (day)	10.0 (9.0, 12.0)	10.0 (9.0, 12.0)	0.568
Total dose of Gn (IU)	1350.0 (1075.0, 1800.0)	1237.0 (1012.0, 1512.0)	0.002
Initiating dose of Gn (IU)	125.0 (112.5, 150.0)	112.5 (100.0, 137.5)	< 0.001
E_2_ levels on the triggering day (pg/ml)	3498.0 (2504.0, 5360.0)	4718.0 (3001.0, 6724.0)	< 0.001
P_4_ levels on the triggering day (ng/ml)	0.9 (0.6, 1.3)	0.9 (0.6, 1.4)	0.580
LH levels on the triggering day (mIU/ml)	2.8 (1.9, 4.3)	3.1 (1.8, 5.0)	0.472
Endometrial thickness on the triggering day (mm)	10.0 (9.0, 12.0)	10.0 (8.8, 11.0)	0.134
Number of oocytes retrieved	16.0 (11.0, 21.0)	22.0 (13.5, 29.0)	< 0.001
Endometrial thickness on the transfer day (mm)	10.0 (8.0, 11.0)	9.0 (8.0, 10.0)	< 0.001
Embryo stage on the transfer day			< 0.001
Cleavage	123 (62.1)	58 (42.0)	
Blastocyst	75 (37.9)	80 (58.0)	
Number of transferred embryos			0.669
1	33 (16.4)	26 (18.2)	
2	168 (83.6)	117 (81.8)	
Embryo transfer strategies			< 0.001
Fresh embryo transfer	110 (54.7)	47 (32.9)	
Frozen–thawed embryo transfer	91 (45.3)	96 (67.1)	
Clinical pregnancy	137 (68.2)	101 (70.6)	0.625
Ectopic pregnancy	4 (2.0)	5 (3.5)	0.498
Early abortion	12 (6.0)	10 (7.0)	0.702
Ongoing pregnancy	121 (60.2)	86 (60.1)	0.991
Late abortion	5 (2.5)	6 (4.2)	0.536
Premature delivery	31 (16.1)	25 (18.4)	0.581
Multiple births	22 (10.9)	14 (9.8)	0.917
Pregnancy complications	32 (15.9)	16 (11.2)	0.456
Live births after the first embryo transfer	116 (57.7)	80 (55.9)	0.744
Live births after the second embryo transfer	35 (62.5)	15 (39.5)	0.028
Cumulative live births after the two embryo transfers	151 (75.1)	95 (66.4)	0.078

Pregnancy complications: hypertensive disorders in pregnancy, gestational diabetes mellitus and hyperemesis gravidarum.

PCOS, polycystic ovary syndrome; BMI, body mass index; AFC, antral follicle count; FSH, follicle-stimulating hormone; LH, luteinizing hormone; E2, estradiol; P4, progesterone; PRL, prolactin; T, testosterone; HOMA-IR, homeostasis model assessment of insulin resistance; FPG, fasting blood glucose; FIns, fasting insulin; Gn, gonadotropin.

AMH levels may indicate the severity of PCOS to a certain extent and can be affected by the heterogeneity of PCOS. In this study, testosterone levels, HOMA-IR values and fasting insulin levels were found to be significantly different in the subgroups with AMH levels of 12 ng/mL as the turning point, which may influence the relationship between AMH and LBR. Notably, patients with higher AMH levels had higher testosterone levels but lower HOMA-IR levels (**Table 1**).

### Correlation between AMH and LBR

AMH levels were adversely associated with LBRs in group C, with or without adjusting for age, BMI, AFCs and baseline FSH and progesterone levels [crude OR 0.881 (0.781–0.994), *P = 0.039* versus adjusted OR 0.796 (0.653–0.971), *P = 0.024*, respectively] (**Table 2**). LBRs decreased with an increase in AMH levels during the second ET cycle [crude OR 0.904 (0.828–0.986), *P = 0.022* versus adjusted OR 0.845 [0.754–0.946], *P = 0.004*, adjusted for age, BMI, AFCs and baseline FSH and progesterone levels) (**Table 3**). In addition, high AMH levels (>12 ng/mL) indicated a high risk of OHSS in group A [crude OR 3.02 (1.03–8.88), *P = 0.045* versus adjusted OR 3.42 (1.1–10.65), *P = 0.034*, adjusted for age and BMI].

**Table 2:** Odds ratio between serum AMH levels and live birth rates in group C.

	Crude odds ratio	Adjusted odds ratio (I)	Adjusted odds ratio (II)
AMH (ng/mL)	0.881 (0.781–0.994)	0.848 (0.733–0.982)	0.796 (0.653–0.971)
*P*-value	0.039	0.028	0.024
Patients grouped based on AMH levels of 12 ng/mL			
1≤AMH<12	1	1	1
12≤AMH<25	0.239 (0.071–0.806)	0.16 (0.039–0.659)	0.086 (0.014–0.527)
*P*-value	0.021	0.011	0.008

Group C: Patients who underwent frozen–thawed embryo transfer after the failure of live birth after the first fresh embryo transfer. Adjusted odds ratio (I): adjusted for age and BMI. Adjusted odds ratio (II): adjusted for age, BMI, AFCs and baseline FSH and P levels.

FET, frozen–thawed embryo transfer; ET, fresh embryo transfer; BMI, body mass index; AFC, antral follicle count; FSH, follicle-stimulating hormone; P, progesterone.

**Table 3:** Odds ratio between serum AMH levels and live birth rate after the second embryo transfer cycles.

	Crude odds ratio	Adjusted odds ratio (I)	Adjusted odds ratio (II)
AMH (ng/mL)	0.904 (0.828–0.986)	0.893 (0.815–0.979)	0.845 (0.754–0.946)
*P*-value	0.022	0.015	0.004
Patients grouped based on AMH levels of 12 ng/mL			
1≤AMH<12	1	1	1
12≤AMH<25	0.391 (0.168–0.912)	0.361 (0.15–0.867)	0.217 (0.074–0.635)
*P*-value	0.030	0.023	0.005

Adjusted odds ratio (I): adjusted for age and BMI. Adjusted odds ratio (II): adjusted for age, BMI, AFCs and baseline FSH and P levels.

BMI, body mass index; AFC, antral follicle count; FSH, follicle-stimulating hormone; P, progesterone.

### Turning point of the correlation between AMH and LBR

**Figure 2:**
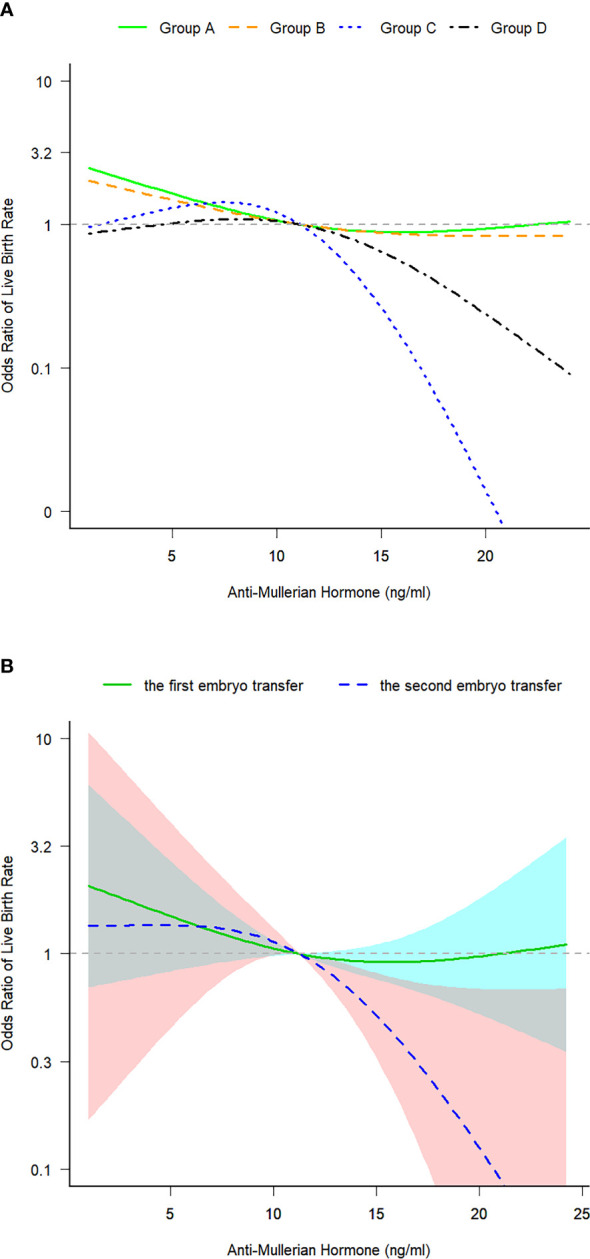
**(A)** Curve fitting for examining the correlation between serum anti-Mullerian hormone levels and live birth rates in the four groups. **(B)** Curve fitting for examining the correlation between serum anti-Mullerian hormone levels and live birth rates after the first and second embryo transfer cycles. Group A: Patients who underwent the first fresh embryo transfer. Group B: Patients who underwent the first frozen–thawed embryo transfer. Group C: Patients who underwent frozen–thawed embryo transfer after the failure of the first fresh embryo transfer. Group D: Patients who underwent frozen–thawed embryo transfer after the failure of the first frozen–thawed embryo transfer. Patients in all four groups were in their first initiated cycle. The 95% confidence intervals were significant only in group C: The model was adjusted for age, BMI, AFCs and baseline FSH and P levels. ET, fresh embryo transfer; FET, frozen–thawed embryo transfer; CI, confidence interval; BMI, body mass index; AFC, antral follicle count; FSH, follicle-stimulating hormone; P, progesterone.

The turning point of the relationship between AMH and LBR was at approximately 12 ng/mL, that is, the intersection of curve fitting of the four groups (**Figure 2A**). Curve fitting demonstrated the following trend: when AMH levels exceeded 12 ng/mL, LBRs remained stable after the first ET cycle but decreased after the second ET cycle (**Figure 2B**). In the second embryo transfers, compared with the patients whose AMH levels were lower than 12ng/mL, LBRs of the AMH level exceeding 12 ng/mL were reduced (62.5% versus 39.5%, *P = 0.028*) (**Table 1**). After the second ET cycle, LBRs were approximately 61%–78% lower in patients with AMH levels of >12 ng/mL than in those with AMH levels of <12 ng/mL [crude OR 0.391 (0.168–0.912), *P = 0.030* versus adjusted OR 0.217 (0.074–0.635), *P = 0.005*, adjusted for age, BMI, AFCs and baseline FSH and progesterone levels] (**Table 3**).

### LBRs of the four groups

**Figure 3:**
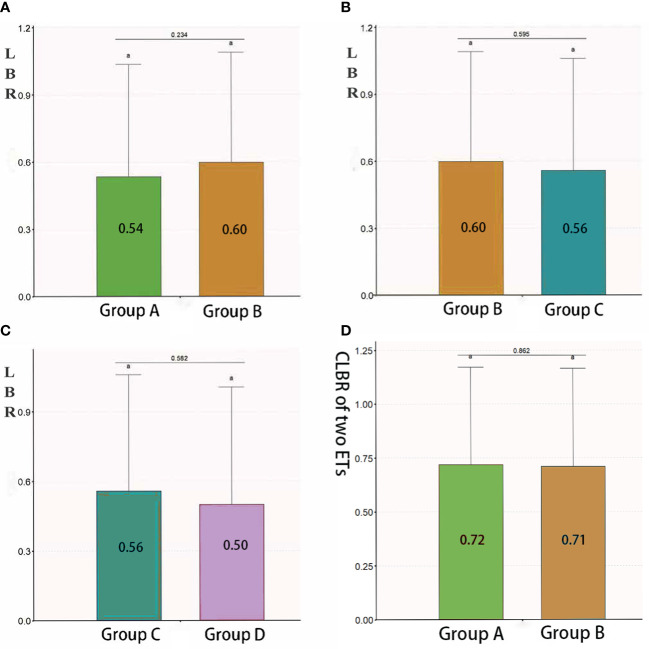
LBR and CLBR in group **(A)**, **(B)**, **(C)** and **(D)**. Patients who underwent the first fresh embryo transfer. Group B: Patients who underwent the first frozen– thawed embryo transfer. Group C: Patients who underwent frozen–thawed embryo transfer after the failure of the first fresh embryo transfer. Group D: Patients who underwent frozen–thawed embryo transfer after the failure of the first frozen–thawed embryo transfer. Groups A and C as well as groups B and D represent two cycles of the same patient. Therefore, they could not be compared with the assumption of independence. When the lowercase letter "a" was observed in both two groups, there were no statistically significant differences between the two groups. LBR, live birth rate; CLBR, cumulative live birth rate; ET, embryo transfer.

The LBRs of groups A, B, C and D were 54%, 60%, 56% and 50%, respectively. The LBR was similar between groups A and B (*P = 0.234*), between groups B and C (*P = 0.595*) and between groups C and D (*P = 0.582*) (**Figure 3**).

### CLBR after two embryo transfer cycles

The TCLBR of all 344 patients was 71.5%, which decreased as AMH levels increased [adjusted OR 0.937 (0.888–0.987), *P = 0.015*, adjusted for age, BMI, AFCs and baseline FSH and progesterone levels). The TCLBR was sufficiently reduced in patients with AMH levels exceeding 12 ng/mL [adjusted OR 0.499 (0.289–0.862), *P = 0.013*] (**Table 4**). The TCLBR was similar between groups A and B (72% versus 71%, respectively, *P = 0.862*) (**Figure 3**).

**Table 4:** Odds ratio between serum AMH levels and cumulative live birth rates after two embryo transfer cycles in all patients.

	Crude odds ratio	Adjusted odds ratio (I)	Adjusted odds ratio (II)
AMH (ng/mL)	0.957 (0.915–1.002)	0.949 (0.905–0.994)	0.937 (0.888–0.987)
*P*-value	0.061	0.027	0.015
Patients grouped based on AMH levels of 12 ng/mL			
1≤AMH<12	1	1	1
12≤AMH<25	0.655 (0.409–1.051)	0.613 (0.378–0.993)	0.499 (0.289–0.862)
*P*-value	0.079	0.047	0.013

Adjusted odds ratio (I): adjusted for age and BMI. Adjusted odds ratio (II): adjusted for age, BMI, AFCs and baseline FSH and P levels.

BMI, body mass index; AFC, antral follicle count; FSH, follicle-stimulating hormone; P, progesterone.

## Comment

### Correlation that is known

Previous studies have suggested that elevated AMH levels are associated with a low LBR after the second ET cycle in women with PCOS (27, 28) and are adversely associated with CLBR (16, 17). In addition, some studies have reported that high AMH levels are associated with adverse pregnancy outcomes, such as a low clinical pregnancy rate (28) and a high preterm birth rate (29). However, some studies have reported that the relationship between AMH and CLBR remains uncertain (28, 30) and that AMH is unlikely associated with adverse pregnancy outcomes (31). This study is a secondary analysis of the association between AMH levels and treatment outcomes of IVF in patients with PCOS. In the first initiated cycle of ET, AMH levels were found to be adversely associated with the LBR of the second ET cycle and the TCLBR.

In addition, an adverse association was observed between AMH levels and LBRs among patients who underwent the second ET cycle with frozen–thawed embryos after the failure of live birth after the first fresh ET.

### Turning point of the correlation

We observed a nonlinear correlation between AMH levels and LBRs in women with PCOS, with a turning point of approximately 12 ng/mL AMH levels. Patients with AMH levels of >12 ng/mL had lower LBRs after the first transfer, much lower LBRs after the second transfer and lower TCLBRs than patients with AMH levels of <12 ng/mL. This finding provides a valuable reference for designing ET strategies based on AMH levels.

### Other results in the context

One of the objectives of this study is to guide clinicians in selecting fresh or frozen–thawed ET based on AMH levels. This study revealed that AMH levels were not associated with the LBR after the first ET cycle. If patients exhibited a high risk of OHSS for the first fresh ET, cryopreservation of embryos was suggested to wait for an optimal transfer condition. The number of patients with AMH levels of >12.0 ng/mL who underwent the first transfer cycle with frozen–thawed embryos was significantly higher than that of patients who underwent the first transfer cycle with fresh embryos. High AMH levels (>12 ng/mL) were correlated with a high risk of OHSS in the first fresh ET cycle. However, LBRs were similar between groups A and B, indicating that AMH levels cannot serve as a criterion for choosing between fresh and frozen–thawed embryos. In addition, LBRs were similar after the first and second frozen–thawed ET cycles (group B versus group C). In summary, if serum AMH levels before the first initiated cycle are <12 ng/mL, selecting fresh embryos for the first transfer is recommended for patients with PCOS, which may save the waiting time for the transfer.

### Clinical and research implications

The present study employed curve fitting to identify the turning point of the relationship between AMH levels and LBRs, thus providing a valuable reference for subsequent research. Considering the similarity of turning points in the same disease, we believe that grouping subjects based on the turning point is more reasonable in analysis compared to traditional quartile grouping, which is also a highlight of the present study.

To improve PCOS patients’ ART live birth rate is a challenge to ART clinicians. The basic conditions of patients requiring the second ET cycle are very likely worse than those of patients with a successful first transfer. The primary pathological mechanisms underlying PCOS may include hyperandrogenemia and insulin resistance (IR). As an auxiliary diagnostic indicator of PCOS, AMH is associated with hyperandrogenemia and IR. Basic interventions such as pre-transfer lifestyle modifications, anti-androgenic therapy and improving IR are recommended for patients with high AMH levels, especially those with the first transfer failure. It is also recommended that to access the risk of PCOS women with high AMH levels before ART treatment to develop appropriate individualized treatment protocols and implement prenatal and follow-up examinations throughout pregnancy. AMH levels play an important role in clinical consultation, which can be used to estimate pregnancy outcomes effectively and inform patients regarding the second ET in advance.

### Limitations and suggestions

This study has some limitations that should be acknowledged. The results of this study cannot be generalized. The inclusion of a limited number of samples from a single center may have led to bias. Smaller sample sizes can deal with only a few confounders, which may result in bias if important confounders are not included in the analysis. In addition, the results may differ if data from other centers are used for analysis. The impact of small sample size can be reflected in parameter estimates. For TCLBR (**Table 3**), the 95% CIs of crude and adjusted odds ratios for variable AMH grouped by 12 ng/ml appeared wilder owing to limited observations in the group with AMH levels of ≥12 and <25 ng/mL. If more observations are recorded in this group, variances may decrease and 95% confidence intervals may be narrower, resulting in smaller *P* values, even for crude odds ratios. Therefore, obtaining representative randomized samples and maintaining a sufficient sample size may help to achieve unbiased results in future studies. The treatment protocol has been described in detail so that other centers can reproduce the findings of this study.

Furthermore, some potential unadjusted confounders may have altered the results of this study. AMH levels were found to be significantly higher in patients who underwent frozen–thawed ET than in those who underwent fresh ET, indicating that the selection of fresh or frozen–thawed embryos may represent a confounder interacting with AMH levels. The important confounders in this study include estrogen and progesterone levels, endometrial thickness, the number of blastocysts formed, and the number of oocytes retrieved (**Table 1**). Baseline covariates were statistically adjusted to ensure outcome validity to the highest extent. However, covariates that changed with baseline characteristics and the dose of medication in the initiated cycle were not adjusted. Future studies should be appropriately designed to focus on the main factors contributing to the decrease in TCLBR and LBR after the second embryo transfer cycle in patients with high AMH levels and to verify the findings of this study.

In this study, the number of patients who completed all possible ET cycles was limited, primarily owing to the lack of embryos and personal reasons. Consequently, we analyzed the CLBR after two ET cycles, with the interval between the two cycles being <1 year. Moreover, the data were outmoded (most recent data collection in 2018), considering that the main article (32) has been published and this study represents a secondary analysis. We will analyze the updated data of CLBR resulting from completed ET cycles in future studies.

## Conclusions

Among PCOS patients high AMH level (>12 ng/ml) is found to be associated with low TCLBR and low LBR of the second embryo transfer cycles. The results provide limited clinical inferences and warrant further investigation.

## Data availability statement

The datasets presented in this article are not readily available because the data are not publicly available due to ethical restrictions. Requests to access the datasets should be directed to FL, liushine2006@163.com.

## Ethics statement

The studies involving human participants were reviewed and approved by the Medical Ethics Committee of Guangdong Women and Children Hospital. The ethics committee waived the requirement of written informed consent for participation.

## Author contributions

NS, JZ and FL contributed to conception and design of the study. CH, SW, XZ and LL organized the database. NS and JZ performed statistical analyses. NS, JZ and YZ interpreted the data. JZ, MX wrote the manuscript. NS, YZ and FL contributed to the critical revision of the article. All authors contributed to the article and approved the submitted version.
